# Endocytosis and Trafficking of Natriuretic Peptide Receptor-A: Potential Role of Short Sequence Motifs

**DOI:** 10.3390/membranes5030253

**Published:** 2015-07-03

**Authors:** Kailash N. Pandey

**Affiliations:** Department of Physiology, Tulane University School of Medicine, New Orleans, LA 70112, USA; E-Mail: kpandey@tulane.edu; Tel.: +1-504-988-1628; Fax: +1-504-988-2675

**Keywords:** atrial natriuretic peptide, natriuretic peptide receptors, guanylyl cyclase, internalization, receptor trafficking, short sequence motifs

## Abstract

The targeted endocytosis and redistribution of transmembrane receptors among membrane-bound subcellular organelles are vital for their correct signaling and physiological functions. Membrane receptors committed for internalization and trafficking pathways are sorted into coated vesicles. Cardiac hormones, atrial and brain natriuretic peptides (ANP and BNP) bind to guanylyl cyclase/natriuretic peptide receptor-A (GC-A/NPRA) and elicit the generation of intracellular second messenger cyclic guanosine 3',5'-monophosphate (cGMP), which lowers blood pressure and incidence of heart failure. After ligand binding, the receptor is rapidly internalized, sequestrated, and redistributed into intracellular locations. Thus, NPRA is considered a dynamic cellular macromolecule that traverses different subcellular locations through its lifetime. The utilization of pharmacologic and molecular perturbants has helped in delineating the pathways of endocytosis, trafficking, down-regulation, and degradation of membrane receptors in intact cells. This review describes the investigation of the mechanisms of internalization, trafficking, and redistribution of NPRA compared with other cell surface receptors from the plasma membrane into the cell interior. The roles of different short-signal peptide sequence motifs in the internalization and trafficking of other membrane receptors have been briefly reviewed and their potential significance in the internalization and trafficking of NPRA is discussed.

## 1. Introduction

**Figure 1:**
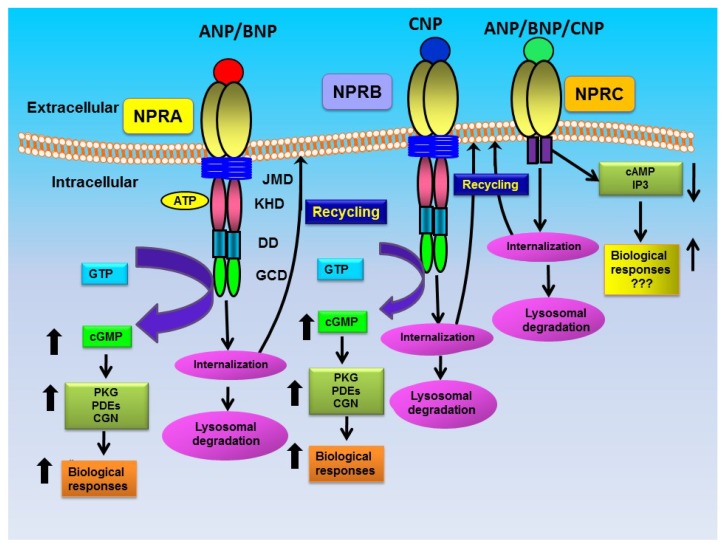
Diagrammatic representation of ligand-dependent activation and post-binding events of NPRA, NPRB, and NPRC: ANP binding activates NPRA, which leads to enhanced production of second messenger cGMP. The bound ligand-receptor complexes of NPRA are internalized into the intracellular compartments and a large proportion of ligand-bound receptors are degraded in the lysosomal compartments, while a small population of internalized receptors recycles back to the plasma membrane. An increased accumulation of intracellular cGMP activates cGMP-dependent protein kinase (PKG), which plays a critical role in ANP-dependent biological responsiveness. The second messenger, cGMP can also activate phosphodiesterases (PDEs) as well as cGMP-gated ion channels to activate ANP-dependent cellular and physiological functions. CNP activates NPRB, which is also internalized, degraded in lysosomes, and recycled back to the cell surface. Ligand- binding to NPRC is suggested to lower cyclic adenosine 3',5'-monophosphate (cAMP) levels and to increase inositoltrisphosphate (IP_3_) in target cells. The bound ligand-receptor complexes of NPRC are internalized, degraded in the lysosomal compartments, and recycle back to the membrane [35] JMD, juxtamembrane domain; KHD, kinase like homology domain; DD, dimerization domain; GCD, guanylyl cyclase catalytic domain.

Atrial natriuretic peptide (ANP) belongs to the natriuretic peptide (NP) hormone family and exerts natriuretic, diuretic, vasorelaxant, antiproliferative, and anti-inflammatory responses, largely directed to the reduction of blood pressure and blood volume [1,2,3]. Two other related peptide hormones, namely brain natriuretic peptide (BNP) and C-type natriuretic peptide (CNP) were later discovered, which also display cellular and physiological responses similar to ANP. The biological actions of these peptide hormones are triggered by their interaction with highly specific NP receptors (NPRs). Three subtypes of NPRs have been characterized and cloned, namely natriuretic peptide receptor-A, -B, and -C; which are designated as NPRA, NPRB, and NPRC, respectively. Both NPRA and NPRB consist of guanylyl cyclase (GC) catalytic domains, which catalyze the formation of intracellular second messenger cGMP from GTP, and are also referred to as GC-A/NPRA and GC-B/NPRB, respectively [4,5,6]. However, NPRC does not contain GC domain and by default has been named as clearance receptor [7,8]. As indicated in Figure 1, ANP and BNP activate NPRA, which produces the intracellular second messenger cGMP [4,5,6,7,8,9,10,11]. Similarly, CNP activates NPRB, which also generates intracellular cGMP [10,11]. All three peptide hormones (ANP, BNP, and CNP) bind to NPRC, which does not produce cGMP. Understanding the intricacies of NPRA signaling is considered to be of pivotal importance to delineate both receptor biology and the disease states, for example hypertension and cardiovascular disorders, which may arise from abnormal hormone-receptor interplay [12,13,14]. It is believed that binding of ANP to extracellular domain of NPRA induces a conformational change in receptor molecule and transmits information to GC catalytic domain; however, the intrinsic mechanism of this activation is not yet clearly understood. Studies, utilizing cultured cells *in vitro* and *Npr1* (coding for GC-A/NPRA) gene-targeted mouse models *in vivo*, have revealed a better understanding of the normal and abnormal control of cellular and physiological functions of NPRA [9,12,13,15,16,17,18,19]. There have been much progress on the biological functions of NPs and their receptors, including cardiovascular, renal, endocrine, neuronal, and skeletal homeostasis; nevertheless, in-depth studies are needed to completely understand the molecular targets in both normal and disease states.

It has been previously suggested that NPRA is a dynamic cellular macromolecule that traverses different subcellular compartments throughout its life-cycle [20,21,22,23,24]. Evidence suggests that after internalization, a large population of ANP/NPRA ligand-receptor complexes are degraded in lysosomes and a small population of receptor recycles back to the plasma membrane [24,25,26]. The cellular life-cycle of NPRA in the context of internalization, recycling, and metabolic processing has opened a new area of signaling mechanisms of GC-coupled receptors. It is believed that internalization of receptors is usually carried out by clathrin-coated vesicles formed on the plasma membrane, and seems to function in a small-peptide sequence-dependent manner. The targeting and sorting of individual membrane receptor and/or protein is directed by their intrinsic sequence-based signal motifs in the endocytic pathways [27,28,29,30,31,32,33,34].

The carboxyl-terminal domains of transmembrane receptors play pivotal roles in mediating the adaptive changes, which accelerate their internalization, trafficking, and subcellular distribution from the cell surface into cell interior [21,27,33,36,37,38,39,40,41,42,43]. The cytoplasmic domains of various membrane receptors are required for internalization, sorting, down-regulation, and desensitization processes. During the process of receptor internalization, endocytic network facilitates the redistribution of bound ligand-receptor complexes (cargo) through diverse subcellular compartments [44,45,46,47]. The complex arrays of routing and trafficking decisions are directed by a set of sorting-signal sequence motifs in the carboxyl-terminal domains of the membrane receptors. The bound ligand-receptor complexes at the plasma membrane, are rapidly internalized through coated pits and vesicles [13,22,31,43,48,49,50,51,52]. The cargo (ligand-receptor complex) is packed into clathrin-coated vesicles to facilitate the process of endocytosis [32,40,49,53]. Subsequently, receptor can travel to early endosomes, late endosomes, and then to lysosomes. Alternatively, receptor could be destined to go to the endosome or plasma membrane involving *trans*-Golgi network (TGN). The intact ligand can rebind to recycled receptor on the plasma membrane and reenter the cell via a repeated process of internalization, termed as retroendocytosis [25].

## 2. Structural Topology and Internalization of NPRA

The amino acid sequence deduced from the primary nucleotide sequence of cDNA revealed that NPRA contains at least four distinct domains, consisting of ligand-binding domain, transmembrane region, protein kinase-like homology domain (protein-KHD), and GC catalytic domain. The amino acid sequences in these regions are conserved across the species among humans, mice, and rats [5,54,55]. The primary structural topology of NPRB is also similar to overall domain structure of NPRA [56]. The nucleotide sequence of *Npr1* is essentially similar among the species, containing 22 exons interrupted by 21 introns [57]. The amino-acid sequence comparisons indicated that a 62% sequence identity exists between NPRA and NPRB, with the intracellular regions appearing to be more highly conserved than the extracellular domains of both receptors. The extracellular ligand-binding domains of NPRA and NPRB show a greater homology to NPRC, which contains a 35-amino acid short cytoplasmic tail and does not consist of protein-KHD and GC catalytic domain [7]. The NPRA mediates most of the known biological actions of ANP and BNP with major cellular and physiological responsiveness, which is mimicked by cGMP and its cell permeable analogs [3,13,21,58,59,60,61]. The protein-KHD of NPRA and NPRB contains an approximately 280-amino acid region that immediately follows the transmembrane spanning domain and is more closely related to protein tyrosine kinases [5,62]. It has been suggested that protein-KHD exerts an important mediatory role in transducing ligand-induced signals to activate GC catalytic domain of receptor [63,64,65,66,67]. GC catalytic domain of NPRA contains an approximately 250-amino acid region that constitutes the catalytic active site of the GC-coupled receptors [26,68,69,70,71]. The transmembrane GC receptors contain a single cyclase catalytic site per polypeptide molecule, however, based on structural modeling data, two polypeptide chains are required to dimerize and activate the receptor molecules [72,73,74,75,76,77]. The crystal structure of the extracellular ligand-binding domain of NPRA has been shown to contain two possible dimer pairs, head-to-head and tail-to-tail, respectively, associated through the membrane-distal and membrane-proximal subdomains. However, a tail-to-tail dimer of NPRA has been proposed [75,76,78]. The crystal structure of NPRC suggests that it is dimerized in a head-to-head configuration bound with ligand [79,80]. It has been indicated that a head-to-head dimer represents the latent inactive state, while a tail-to-tail dimer could represent hormone-activated state of the receptor molecule [81]. Later, it was reported that ANP binding stabilizes NPRA dimer with more stringent spacing at the interface [82]. The studies of site-directed mutagenesis and chemical modification experiments have suggested that a head-to-head structure reflects the physiological dimmer structure of GC-coupled receptors [76,78].

### 2.1. Ligand-Mediated Internalization

Initial studies have shown that after ANP binding, ligand-receptor complexes are internalized into cell interior [20,83,84]. The internalization of ANP also occurs through NPRC [35,85,86,87,88,89,90,91,92]. Studies using Leydig tumor (MA-10) cells containing a high-density of endogenous NPRA and human embryonic kidney-293 (HEK-293) as well as COS-7 cells expressing recombinant NPRA, established that ligand-receptor complexes of ANP-NPRA are rapidly internalized in a ligand-dependent manner and redistributed intracellularly in intact cells [20,21,24,71,93]. Those previous finding demonstrated that after ligand-binding, NPRA is internalized at physiological temperatures and both degraded and intact ligands are released into culture medium [20,22,23,25,26]. Distribution of ^125^Iodine-labeled-ANP (^125^I-ANP) radioactivity on the cell surface, in the intracellular compartments, and in culture medium provided a dynamic equilibrium of receptor-mediated ^125^I-ANP uptake, degradation, and extrusion. A major portion of the internalized ^125^I-ANP was released into culture medium, which consisted of approximately 75% degraded products and about 25% intact ligand [20,22,26,93]. The release of both degraded and intact ligands was blocked by lysosomotropic agents, ammonium chloride (NH_4_Cl_2_) and chloroquine [20,94]. Those previous studies suggested that most of the internalized ^125^I-ANP was processed through the lysosomal degradative compartments in intact cells; however, a population of internalized receptors recycled back to the plasma membrane (Figure 1). After internalization, most of the endocytosed ligand was degraded in the lysosomes and then released into culture medium, however, approximately 25% of ligand-receptor complexes escaped the lysosomal degradative pathway and extruded intact outside the cell [25,26,41,77]. Using an antibody-tracking method, one study has indicated that in specific cells both NPRA and NPRC were internalized in a ligand-independent manner [95]. Since, antibody-tracking method could determine the internalization kinetics only qualitatively; additional studies are needed to support these conclusions.

Previous studies have suggested that homeostatic regulation of NPRA and its cellular sensitivity to ANP are dependent on a dynamic equilibrium of endocytosis and intracellular processing events [25,94]. The rates of internalization and degradation of ^125^I-ANP-bound NPRA were markedly diminished in the presence of lysosomotropic inhibitors, chloroquine and NH_4_Cl_2_ as well as energy-depleter dinitrophenol [19,24]. Both chloroquine and NH_4_Cl_2_ are known to inhibit the lysosomal degradation, while dinitrophenol disrupts the energy-dependent intracellular trafficking of membrane receptors [77,93,94,96,97,98,99,100]. The kinetics of receptor binding in trypsin-treated and solubilized cell extract have demonstrated that most of the receptors were present on the plasma membrane and approximately 10% receptor population was assigned to the preexisting intracellular pool [25,26]. Usually, chloroquine, NH_4_Cl, nigericin, and monensin and the metabolic inhibitor dinitrophenol (depletes cellular ATP), have been found to disrupt the internalization as well as recycling of NPRA [20,23,25,26]. However, ATP is not required for the internalization of insulin receptor but needed for internalization of epidermal growth factor (EGF) receptor [96,101,102]. The internalized hormone-receptor complexes of low-density lipoprotein (LDL) enter the acidic vesicular compartments where ligand dissociates from the receptor, which recycles back to the plasma membrane and ligand is degraded in the lysosomal compartments [103]. Similarly, after endocytosis of NPRA, some of the internalized pool of receptors seem to be diverted to the degradative pathway, while remainder of the receptors enter the regulated recycling pathway [20,23,94]. Certain class of receptors may regulate their own biosynthesis involving intracellular signals, generated during the ligand-dependent receptor internalization and trafficking events. The ligand-stimulated internalization and partial degradation of γ-aminobutyric acid (GABA) receptors both seem to enhance and repress receptor gene expression [104,105,106]. The intriguing finding was that agonist-dependent endocytosis of ß2-adrenergic receptor is a necessary step in the activation of its mitogenic signals [107]. However, this functional significance correlating with the ligand-dependent internalization of NPRA are not yet known.

### 2.2. Down-Regulation and Metabolic Degradation

Ligand-dependent down-regulation of receptor involves the physical loss of cell surface receptor molecules rather than their redistribution into the subcellular compartments. The internalization of NPRA seems to be critical for its down-regulation process [23,25,94]. After binding of ANP to NPRA, ligand-receptor complexes are internalized, sequestered into intracellular compartments, and degraded products are released into the culture medium [93,94]. The pretreatment of HEK-293 cells with unlabeled ANP causes a substantial decrease in ^125^I-ANP binding of NPRA [25,94]. Ligand-dependent down-regulation of NPRA has also been reported in primary bovine aortic endothelial cells, immortalized HeLa cells, and 293 T cells stably overexpressing this receptor protein [108]. The mechanisms regulating the down-regulation of receptors, involve complete removal of ligand-bound receptors from the plasma membrane into cell interior. There could be several components of this phenomenon, including ligand-dependent receptor internalization, ligand degradation, and recycling of receptor back to the plasma membrane. Alternatively, both the receptor and ligand can be degraded in lysosomal compartments. If a receptor recycles to plasma membrane, the internalization can be compensated to some extent by reappearance of endocytosed receptors on the cell surface. Thus, down-regulation can be delayed until receptors begin to be degraded in the lysosomal compartments. Essentially, down-regulation of NPRA may result in a loss of receptor from the cell surface by means of an enhanced rate of receptor internalization and degradation. Receptors that are metabolically processed and degraded after internalization can have important physiological and pathophysiological implications, including an effect on promoting ligand-receptor internalization that would lead to the degradation of both ligand and receptor molecules in the lysosomes. In a process of receptor down-regulation, an increased rate of degradation can exceed the rate at which receptors are replaced by *de novo* synthesis, so that a total number of receptors are correspondingly reduced on the cell surface [25,26,109]. The receptor-mediated endocytosis of ANP/NPRA complexes may involve a number of sequential sorting steps through which ligand-receptor complexes could be either eventually degraded, recycled back to the cell surface, or released into the cell exterior [21,22,23,25]. These events may take place sequentially as follows: (i) binding of ligand to cell surface receptor on the plasma membrane, may lead the accumulation of ligand-receptor complexes into coated pits or vesicles, (ii) the coated pits fuse with early endosomes, which largely deliver cargo into the lysosomes, and (iii) early endosomes may also deliver the receptor to recycling endosomes, which may recycle the receptor back to the plasma membrane. Our previous findings have suggested that after internalization, ANP/NPRA complexes enter into a degradative pathway through which 75% of internalized ANP is processed in the lysosomes and 25% ligand is released as intact molecules through a recycling pathway, which may rebind to new receptor molecules [20,25].

The degradative processing of ANP-NPRA seems to be similar to several other membrane receptors, including low density lipoprotein (LDL) receptor in human fibroblasts [110,111], insulin receptor in adipocytes [112,113,114], and thyrotropin hormone receptor in GH3 cells [115]. However, the degradation of asialoglycoprotein- receptor complexes in hepatoma cells, is not observed until about 30 min after the endocytosis [116]. Similarly, degradation of EGF-receptor complexes, was not detectable for at least 20 min in hepatocytes [117]. There seems to be multiple pathways leading to the eventual metabolic turnover of ligand-receptor complexes, involving different intermediate vesicles for the transfer of ligand to the site of degradation. It is possible that there is a single metabolic pathway composed of several distinct processing steps, which should be specific for a distinct ligand-receptor complex. Dual pathways for the intracellular processing of ligand-receptor complexes have also been suggested for insulin and EGF receptors [99,118,119,120,121]. Similarly, the degradative processing of NPRA without exerting a deleterious effect on the retroendocytotic pathway is intriguing [25,94]. Those previous studies provided the notion that after internalization, ligand-receptor complexes can recycle through chloroquine-insensitive pathway and finally be degraded via chloroquine-sensitive lysosomal compartments [24,25]. Accumulating evidence suggests that several types of ligand-receptor complexes, including for EGF, insulin, and asialoglycoprotein, also recycle through the chloroquine-insensitive pathway [31,114,119,120,122,123].

### 2.3. Inactivation or Desensitization

It is believed that desensitization of receptor occurs intracellularly and that the inhibition of internalization may prevent this process. Desensitization of receptor can be defined as a phenomenon whereby the function of a receptor is lost or inactivated. Degradation, on the other hand, has been considered as the removal or actual loss of receptor from the cell surface by internalization followed by lysosomal proteolysis. Inactivation can precede degradation or both events may occur simultaneously. In the metabolic processing studies of NPRA, we have used ANP-binding parameters as an index of receptor activity [25,26]. ANP-induced receptor internalization might be an invaluable experimental tool for elucidation of NPRA inactivation or desensitization. A change in the state of receptor phosphorylation has also been implicated as a marker in the process of receptor inactivation or desensitization [10]. Desensitization does not require large-scale physical removal of receptors from the plasma membrane, however, is probably achieved by a combination of both receptor phosphorylation and degradation processes. The inactivation and/or desensitization of NPRA occur by both ANP-dependent dephosphorylation as well as the internalization of receptor molecules [25,26,124]. The exact mechanism of dephosphorylation-dependent desensitization of NPRA is not clear. The previous studies have suggested that phosphorylation of NPRA seems to occur in various cells and tissues [63,125,126,127,128,129]. However, the nature of a specific protein kinase, which phosphorylates NPRA, has yet to be determined.

### 2.4. Role for microRNA Interference and Endocytosis

RNA interference (RNAi) is a powerful means to suppress gene expression in mammalian cells [130]. Endogenously expressed small single-stranded RNA sequences of approximately 22 nucleotides direct gene silencing, through components that share RNAi pathway [131]. The unique structural feature of microRNAs (miRNAs) is their initial synthesis as a long primary transcript, which are processed by a nuclear enzyme Drosha into approximately 70 nucleotide stem-loop hairpin RNAs precursor molecules [132]. Primary-miRNA is exported from the nucleus to the cytoplasm by exportin-5, a nuclear transport receptor and processed by Dicer into approximately 22 nucleotides of mature miRNA, which is subsequently incorporated into miRNA-containing RNA-induced silencing complex [133,134,135,136]. Target cleavage can be artificially induced by altering the miRNA sequence to obtain complete hybridization [137,138,139]. Many natural miRNA hairpins exist in clusters of multiple copies, which regulate gene expression by mRNA cleavage or translational repression. We have utilized RNA interference to silence the expression of *Npr1*, providing a novel system to study the internalization and trafficking of NPRA in intact cells [140]. The miRNA-mediated small interfering RNA (siRNA) elicited functional gene-knockdown of NPRA in stably transfected HEK-293 cells expressing a high density of recombinant receptors. *Npr1* miRNA caused a drastic reduction in the internalization of ligand-receptor complexes. Only 10%–12% of receptor population was localized in the intracellular compartments of micro-RNA silenced cells as compared to 70%–80% in control cells [140]. ANP-stimulated intracellular accumulation of cGMP and GC activity of NPRA were drastically reduced in *Npr1* miRNA-expressing cells by 90%–95% as compared with control cells. Cells expressing *Npr1* miRNA showed a very low receptor density on the plasma membrane and a reduced accumulation of intracellular cGMP compared with control cells indicating that polycistronic expression of artificial multi-miRNA silenced *Npr1* gene function. It was implicated that post-transcriptional silencing of *Npr1* gene used by polycistronic miRNA, reduced the receptor expression and intracellular accumulation of second messenger cGMP.

### 2.5. Clathrin *Versus* Caveolae-Mediated Trafficking of Membrane Receptors

**Figure 2:**
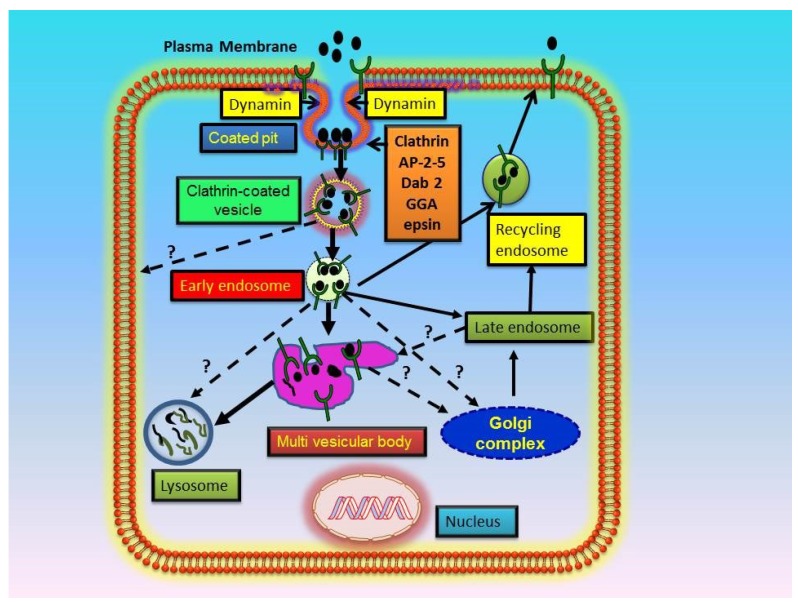
Schematic representation of ligand-mediated receptor endocytosis and trafficking pathways: Ligand-binding to cell surface receptors leads to a selective recruitment of ligand-receptor complexes (cargo) into clathrin-coated pits. The coated pit represents a small area of the plasma membrane, which invaginates and pinches off into vesicles in the cytoplasmic compartments. The coated vesicles trigger the recruitment of APs for example AP-1, AP-2, AP-3, AP-4, and AP-5 as well as other interacting protein molecules such as disabled-2 (Dab-2) and Golgi-localizing gamma adaptor homology domain binding protein (GGA). The clathrin-dependent routes require dynamin to achieve the fission of membrane invaginations and subsequent internalization of vesicle into the cell interior. The ligand-receptor complexes within the vesicles entering via clathrin pathway are usually directed to early endosomes. Within the endosomes, ligand-receptor complexes are sorted and directed to various subcellular compartments, where the internalized receptors are either routed to degradative compartments such as late endosomes, multi vascular bodies, and/or lysosomes, or recycled back to the plasma membrane via recycling endosomes. Alternatively, the internalized cargo may be sequestered in endosomes for a longer period of time and continue to spark signaling events. Some early and late endosomes also contain membrane structures in the lumen, which are referred to as multi-vesicular bodies (MVBs). The endosomes and lysosomes can also transmit and receive cargo from trans-Golgi network (TGN) involving vesicular intermediate carriers. The key proteins involved in the trafficking of molecules at different locations may include: AP-1, AP-2, AP-3, AP-4, AP-5, Dab-2, and GGA.

Clathrin-mediated internalization is regarded as an established mechanism for transport of a large number of membrane receptors and proteins from the plasma membrane to intracellular compartments [31,32,43,141,142,143,144]. Interestingly, clathrin-dependent endocytosis is considered a major pathway for concentrative uptake and internalization of cargo or ligand-receptor complexes inside the cell [50,51]. The coated vesicles or pits originate by deepening invagination and dissociation from the plasma membrane, which produce a clathrin-coated vesicle, involving the mechanochemical force generated by dynamin [40,49,53,145,146,147]. It has been suggested that clathrin-coated vesicle formation involves at least five stages including initiation of invagination or pit formation, cargo selection, coat assembly, scission, and uncoating [46,148]. It is believed that clathrin does not bind directly to either the membrane or the cargo complex; rather, it accelerates the recruitment of adaptor proteins (APs) and other accessory proteins such as epsin. The coated vesicles usually give rise to endosomes, recycling endosomes, and/or lysosomes. The membrane receptors with bound ligand within the coated vesicles or pits are rapidly internalized and delivered to the endosomes (Figure 2). In the endosomes, ligand-receptor complexes are either directed to lysosomes, where they are eventually degraded, or receptors can be inserted into the plasma membrane via recycling endosomes, where it can rebind to a new ligand and this phenomenon is referred to a retroendocytosis [25]. The endosomal receptor proteins seem to recycle back to the plasma membrane to maintain a steady-state equilibrium [149]. It is implicated that differential sorting of ligand-receptor complexes between distinct endocytic pathways seems to be a common phenomenon for different types of ligand-receptor complexes [25,26,45,77,150]. The work from our laboratory has indicated that GC-A/NPRA is also internalized involving clathrin-dependent coated vesicles [151,152]. Our unpublished results have indicated that the internalization of NPRA is severely diminished by chlorpromazine and monodensylcadaverine, inhibitors of dynamin, which facilitates clathrin-dependent receptor internalization. Nevertheless, more elaborate studies are needed to clearly demonstrate the role of clathrin-coated pits and adaptor proteins in the internalization of NPRA, NPRB, and NPRC.

Certain membrane receptors travel involving caveolae, which are assembled by the protein caveolin that is constituted in the endoplasmic reticulum and then travels to the plasma membrane. Caveolae participate in the internalization of various types of macromolecules and also function as docking sites for the assembly of intracellular signaling cascades [153,154,155]. A number of cargo molecules are known to be internalized involving caveolae such as transforming growth factor-beta receptor (TGF-βR), ubiquitinated EGF receptor, integrins, adenosine receptors, and glutamate transporters [156,157,158]. Caveolae-dependent endocytic mechanisms also require dynamin in a short peptide sequence-dependent manner; however, there is only limited similarity between clathrin-mediated and caveolae-dependent internalization mechanisms of membrane receptors and proteins.

Caveolae originate from the plasma membrane invaginations and play critical roles in various cell functions for a variety of receptors, channels, and other membrane proteins during internalization, transport, and signaling [159,160]. Caveolae are characterized by flask-shaped plasma membrane invaginations and have been noted on the surface of numerous cell types, including endothelial, vascular smooth muscle, and epithelial cells [159,161]. Usually, caveolae are enriched in cholesterol, sphingolipids, and proteins specifically caveolin 1–3 and cavin 1–4, which serve as sensors of membrane tension [162]. ANP has been shown to enhance transendothelial caveolae-mediated albumin transport by activating NPRA [161]. Those previous findings suggested that the transendothelial vesicular pathway participates in the stimulatory effect of ANP/NPRA system on permeability of albumin in the microcirculation, thereby causing alterations in blood volume. Earlier, it has also been shown that NPRB was localized in cardiac myocyte caveolae [163]. Overexpression of myocyte-specific caveolin3 attenuated cardiac hypertrophy and increased the expression and signaling of both ANP and BNP [164]. However, more studies are needed to define the additional roles caveolae play in the functional aspects of natriuretic peptides and their cognate receptors in target cells. Evidence suggests that Eps15 homology domain-containing 2 (EHD2), a dynamin-related ATPase participates in the remodeling of curved membrane, resulting in caveolae formation [165,166,167]. These authors suggested that EHD2 associates with caveolin, and does not participate in clathrin-mediated endocytosis or endosomal trafficking as previously indicated [168]. After endocytosis, caveolae structures appear to be highly stable compared with clathrin-coated pits [169,170]. Recent studies have suggested the role of a novel protein endophilin-A 2 (ENDO-A2) involving membrane scission in clathrin-independent endocytosis [171,172]. Intriguingly, their studies indicated that in human and other mammalian cell lines, ENDO-A2 specifically participated with early structures involved in the endocytosis independent of clathrin-mediated processes. However, the involvement of caveolin and the role of new protein (ENDO-A2) in the endocytosis and trafficking of membrane receptors, including the natriuretic peptide receptors should be the subject of future investigations.

## 3. Internalization and Trafficking of NPRB

Interestingly, GC-B/NPRB has also been shown to internalize and recycle back to the plasma membrane in hippocampus neurons and C6 glioma cells [173]. Those previous studies indicated that trafficking of NPRB occurs in response to CNP-binding and stimulation of receptor-mediated endocytosis. It was suggested that neuronal Ca^2+^ sensor protein, visinin-like protein-1 (VILP-1) mediates the internalization and trafficking of NPRB involving a clathrin-dependent mechanism. However, the heterologous expression of VILP-1 leads to an increased cell surface expression and attenuation of NPRB endocytosis, which was not observed with highly homologous protein VILP-3 [173].

## 4. Role of C-Terminus Domain and Small Peptide Sequence Motifs in the Internalization of Membrane Receptors

The sequential deletion of NPRA at the C-terminus end, significantly reduced the internalization and metabolic processing of ligand-receptor complexes compared with wild-type receptor [23]. The complete deletion of both protein-KHD and GC catalytic domains abolished the internalization of NPRA; however, deletion of 170 amino acids at the C-terminus end of receptor, decreased the internalization by 60% [23]. Those previous findings suggested that specific regions within intracellular domains of NPRA determine the extent of ligand binding, endocytosis, and intracellular sequestration of receptor [23]. Due to sequential deletions of amino acid residues at the C-terminus region of receptor, a large proportion of ligand-receptor complexes did not internalize and remained on the plasma membrane. Interestingly, most of the internalization signals seem to be present in the cytoplasmic domains of membrane receptors capable of endocytosis [22,174,175,176,177]. Evidence suggests that a majority of the receptors that undergo endocytosis contain internalization signal sequence motif in the cytoplasmic region, near the transmembrane domain or C-terminus tail of the receptor molecules. Internalization of numerous membrane receptors is governed by recognition of signal sequence motifs within the cytoplasmic domains, which facilitate receptor endocytosis through clathrin-coated pits or vesicles into the cell interior [39].

The roles of different short peptide sequence motifs in the internalization and trafficking of membrane receptors will be briefly reviewed and their potential roles in the internalization and trafficking of NPRA will be discussed. The small peptide sequence motifs play critical roles in the internalization and trafficking of membrane receptors and often act as sorting signals to direct cargo into vesicles in clathrin-dependent manner [33,38]. A hallmark characteristic feature of endocytic and trafficking signals that distinguishes a particular sequence motif from other sequences, is their presence in the cytoplasmic domains of transmembrane receptors. The small peptide sequence motifs usually constitute short linear arrays of amino acids, which consist of four to seven amino acid residues [27,33,34,38,178]. However, only two or three amino acid residues within the signal motifs usually play a critical role in the internalization process. The functional residues are most likely the bulky hydrophobic amino acids, however, it has been suggested that the charged amino acid residues are also important determinants of the specificity and functional significance in the endocytosis and sorting of membrane receptors. The continuous efforts are being made to identify new sequence motifs for endocytosis and trafficking of various membrane receptors, including NPRA. Thus far, one specific signal sequence motif GDAY in the carboxyl-terminal domain of NPRA has been shown to play roles in the internalization and trafficking of this receptor protein [26]. The identification of short sequence motifs using high performance algorithms through the genome databases should provide additional new signal motifs playing role in receptor internalization [179]. The major types of membrane receptors and proteins with known endocytic and sorting signal sequence motifs are presented in Table 1.

**Table 1:** Selected short-sequence motifs for internalization and trafficking of membrane receptors and proteins.

Membrane Receptor/Protein	Signal Motifs (1-Letter Code)	Amino Acids (3-Letter Code)	Reference
Acetylcholine transporter	DSLL	Asp-Ser-leu-Leu	[180]
Beta-amyloid precursor protein	YENPTY	(Tyr-Glu-Asn-Pro-Thr-Tyr)	[181]
CD3 Chains	[DE]xxxL[LI]	(Asp-Glu-x-x-x-Leu-Iso)	[182]
CD-Mannose-6-phosphate receptor	YKYSKV	(Tyr-Lys-Tyr-Ser-Lys-Val)	[183]
CI-Mannose-6-phosphate receptor	YSKV	(Tyr-Ser-Lys-Val)	[184]
GC-A/natriuretic peptide receptor-A	GDAY	(Gly-Asp-Ala-Tyr)	[26]
Integrin	NPxY	(Asn-Pro-x-Tyr)	[185]
Insulin-like growth factor receptor	YxxPhi	(Tyr-x-x-Leu)	[183,184]
LDL receptor	FDNPVY	(Phe-Asp-Asn-Pro-Val-Tyr)	[186]
LH receptor	GTALL	(Gly-Thr-Ala-Leu-Leu)	[187]
LDL-related receptor	YATL	(Tyr-Ala-Thr-Leu)	[188]
Mannose phosphate receptor	FENTLY	(Phe-Glu-Asn-Thr-Leu-Tyr)	[183,184]
Platelet activating factor receptor	[YF]xNPx[YF]	(Tyr-Phe-x-Asn-Pro-Tyr-Phe)	[189]
Protease-activated receptor-1	YKKL	(Tys-Lys-Lys-Leu)	[190]
P2x receptor (ATP-gated ion channel)	YEQGL	(Tyr-Glu-Gln-Gly-Leu)	[191]
Transferrin receptor	YTRF/Q	(Tyr-Thr-Arg-Phe/Leu)	[183,184]
T-cell receptor	YQPL	(Tyr-Gln-Pro-Leu)	[184]
Glutamate receptor	YWL	(Tyr-x-Leu)	[192]

A large number of short sequence motifs usually contain tyrosine or phenylalanine residues, followed by hydrophobic or aromatic residues. Some sequence motifs also contain acidic residues in conjunction with required tyrosine. Certain membrane receptors make use of dileucine-type of signal motifs. GC-A, guanylyl cyclase-A; GLUT4, glucose transporter 4; LDL, low density lipoprotein; LH, leutinizing hormone; TGN, *trans*-Golgi network; x, refers to any amino acid residue; Phi, refers to hydrophobic amino acid residue.

### 4.1. GDAY Motif and Internalization of Membrane Receptors

A previous study from our laboratory has demonstrated that GDAY (Gly–Asp–Ala–Tyr) motif located in the carboxyl-terminus domain of NPRA plays dual functional roles in the internalization and subsequent recycling processes. The mutations of Gly^920^ and Tyr^923^ residues to alanine in GDAY motif of NPRA, attenuated the internalization of mutant receptor by almost 50% as compared with wild-type (WT) receptor [26]. However, the mutation of Asp^921^ to alanine had only a minimal effect on internalization of NPRA. The regulation of receptor internalization relies largely on residues Gly^920^ and Tyr^923^ and mutation of Asp^921^ to alanine significantly attenuates the recycling of internalized receptor back to the plasma membrane**.** Thus, the functional role of cytoplasmic tail of NPRA is important for receptor internalization, as has been observed in case of thyrotropin-stimulating hormone receptor [193]. Deletion mutations within the carboxyl-terminal region of NPRA also seem to exert a major consequence on its internalization process [23]. The replacement of selected Gly^920^, Asp^921^, and Try^923^ residues with alanine in GDAY motif showed that internalization of mutant receptor was significantly reduced compared with WT receptor [26].

More specifically, residues Gly^920^ and Tyr^923^ constitute important elements in GDAY motif for internalization; however, Asp^921^ provides an acidic environment for efficient recycling of internalized receptors. Two overlapping motifs within the GDAY sequence seem to exert different but specific effects on endocytosis and subsequent trafficking of NPRA in the subcellular compartments [22,26]. It is evident that tyrosine-based GDAY motif modulates early internalization of NPRA, whereas Asp^921^ residue in GDAY sequence seems to mediate recycling or latter sorting of receptor molecules. Similarly, Gly^950^-Pro^951^-Leu^952^-Tyr^953^ motif has been implicated in the internalization of insulin receptor, in which Gly^950^ and Tyr^953^ residues play critical roles in the internalization process [194]. Interestingly, GDAY motif is also present in the NPRB; however, the involvement of GDAY motif in the internalization and trafficking of NPRB remains to be elucidated.

### 4.2. NPXY Motif and Internalization of Membrane Receptors

Initially, FxNPxY was recognized as the first short sequence signal motif in the cytoplasmic domain of membrane receptors, to play roles in the trafficking and sequestration processes [111]. However, NPxY is located in the extracellular domain of NPRA, thus it is not expected to play a role in the internalization and trafficking of this receptor protein. NPxY signal motifs have been shown to mediate the internalization of several membrane receptors and proteins, including beta-1 integrin, megalin, beta-amyloid precursor protein, EGF receptors, and neurotrophin receptors [27,195,196,197,198,199]. The early studies demonstrated that substitution of a cysteine residue for a tyrosine residue in NPxY (Asn-Pro-x-Tyr) motif of LDL receptor in a patient with familial hypercholesterolemia, rapidly abrogated its internalization [186]. The previous studies have also indicated that NPxY motifs recruits clathrin and adaptor proteins and act as cargo recognition sequence for their delivery to endosomes and lysosomes [37,200]. Interestingly, NPxY motifs initially recruit adaptor proteins at the plasma membrane and activate mu2 subunit of AP-2, after which the beta-2 subunit of AP-2 binds clathrin at the cell surface, leading to clathrin-mediated endocytosis [27,37,190,201,202]. The adaptor protein Dab-2 directly interacts with NPxY motifs and leads to clathrin-mediated endocytosis by activating clathrin and AP-2 [203,204]. FDNPVY sequence motif binds to components of clathrin coat and in this context, NPxY residues adopt a beta-turn structure [205,206]. Similarly, endocytosis of beta-amyloid precursor protein is directed by a longer sequence motif, GYENPTY in which the first tyrosine residue seems to play a critical role in the internalization process [207].

It has been suggested that proteins containing phosphotyrosine-binding (PTB) domains, participate in LDL receptor internalization. PTB domains containing Dab-1 and Dab-2 directly bind to FxNPxY sequence motifs located in the members of LDL receptor family [181,185,208]. The overexpression of PTB domain of either Dab-1 or Dab-2 impedes internalization of LDL receptor, leading to accumulation of receptors on the cell surface. Both Dab-1 and Dab-2 contain signal motifs, which bind to clathrin and AP-2 at the carboxyl-terminus of their PTB domains [189,200,203,209,210]. Similarly, Grb-2 has been shown to facilitate internalization of EGF receptor [211]. In the carboxyl-terminal domain of beta-5 integrin, NPxY motifs act as a molecular switch for distinct biological processes of integrin activation, including endocytosis, sorting, and recruitment of the adaptor proteins and clathrin for endocytosis and internalized cargo assembly [45,51,195,212,213]. It should be emphasized that NPxY motif is located in the ligand-binding domain of NPRA (residues Asn^315^-Pro^316^-x-Tyr^318^); however, due to its location in the extracellular domain, it may not participate in the internalization process (Table 2). Most of the signal motifs that direct endocytic processes are located in the carboxyl-terminus region of membrane receptors and interact with the adaptor proteins to facilitate the internalization process.

**Table 2:** A list of putative signal sequence motifs located in the extracellular ligand-binding region, protein kinase-like homology domain, and guanylyl cyclase catalytic domain of natriuretic peptide receptor-A (NPRA).

Signal Motifs	Amino Acid Sequence	NPRA Sequence	Reference
DPxxY	Asp^220^-Phe^221^-x-x-Try^224^	Ligand-binding domain	[5]
YTKL	Try^224^-Thr^225^-Lys^226^-Leu^227^	Ligand-binding domain	[5]
YVFF	Try^264^-Val^265^-Phe^266^-Phe^267^	Ligand-binding domain	[5]
NPxY	Asn^315^-Pro^316^-x-Phe^318^	Ligand-binding domain	[5]
YLEF	Try^317^-Leu^318^-Glu^319^-Phe^220^	Ligand-binding domain	[5]
KKFN	Lys^331^-Lys^332^-Phe^333^-Asn^334^	Ligand-binding domain	[5]
DGLLL	Asp^351^-Gly^352^-Leu^353^-Leu^354^-Leu^355^	Ligand-binding domain	[5]
YLKI	Phe^390^-Leu^391^-Lys^392^-Ile^393^	Ligand-binding domain	[5]
YWPL	Phe^437^-Met^438^-Pro^439^-Leu^440^	Ligand-binding domain	[5]
YGSL	Phe^536^-Gly^537^-Ser^538^-Leu^539^	Protein-KHD	[5]
SLL	Ser^538^-Leu^539^-Leu^540^	Protein-KHD	[5]
KKLW	Lys^694^-Lys^695^-Leu696-Trp^697^	Protein-KHD	[5]
FQQI	Phe^790^-Gln^791^-Gln^792^-Ile^793^	Protein-KHD	[5]
YQIL	Tyr^846^-Gln^847^-Ile^848^-Leu^849^	Protein-KHD	[5]
YTCF	Tyr^901^-Thr^902^-Cys^903^-Phe^904^	GC Catalytic domain	[5]
GDAY	Gly^920^-Asp^921^-Ala^922^-Try^923^	GC Catalytic domain	[5,26]
YMVV	Tyr^923^-Met^924^-Val^925^-Val^926^	GC Catalytic domain	[5]
YCLF	Tyr^998^-Cys^999^-Leu^1000^-Phe^1001^	GC Catalytic domain	[5]
YWLL	Tyr^1045^-Trp^1046^-Leu^1047^-Leu^1048^	GC Catalytic domain	[5]

Internalization of platelet-activating factor is also regulated by DPxxY motif [209]. Similarly, the internalization of type-2 vasopressin receptor is enhanced by NPxxY sequence [214]. A common feature of these internalization signal motifs, including NPxY and GDAY, is the presence of a tyrosine residue at the end of tetrapeptide sequence motif [24,26,209]. Moreover, tyrosine residues in mannose-6-phosphate receptor and influenza virus hemagglutinin are also involved in the endocytosis and intracellular trafficking, even though they are not present in the context of NPxY or YxRF consensus motifs. Therefore, if a universal internalization signal exists, it may not be based on a universal amino acid sequence [27,37,188,209,215]. The critical features of the internalization sequences might be their specification of a particular conformation, such as a tight beta-turn in the structure of receptor molecules.

The tyrosine recognition sequence forms a small surface loop but differs in terms of the positioning of tyrosine in the loop structure [27,184,206]. The substitution for Tyr with inactive residues has resulted in the disruption of beta-turn conformation. A similar approach was used to obtain evidence that PPGY sequence in the acid phosphatase cytoplasmic tail, forms a type 1 beta turn with Tyr in the fourth position [216]. Thus, the presence of Tyr in the fourth position of signal motifs seems to be critical in the receptor internalization. The seven transmembrane G-protein-coupled receptors contain homologous motif NPxxY and a mutation to NPxxA resulted in a complete loss of agonist-induced receptor internalization and sequestration [217,218]. The conserved Tyr residue is also required for the internalization of vasopressin receptor [214]. However, there are exceptions to this rule, for example, in YxxF (Tyr-x-Arg-Phe) motif, the critical Tyr residue is included at the amino-terminus first position, which provides general consensus of Yxxphi motifs for internalization of insulin-like growth factor, mannose-6-phosphate, and transferrin receptors [27,184,219]. Tyrosine also seems to exert a basolateral sorting function, where it is shared by distal NPxY motif and by endocytic motif YATL for internalization of LDL receptor-related proteins [220,221]. It has been suggested that YxxL motif functions in the endocytosis of LDL receptor-related proteins [221]. Similarly, a tyrosine-based motif, YWL also functions as an endocytic motif for internalization of NMDA-type glutamate receptor [222].

### 4.3. Dileucine Motifs and Endocytosis of Membrane Receptors

Several dileucine-based motifs, including YGLL, SLL, and YWLL are also located in the protein-KHD and GC regions of NPRA, however, their roles in the internalization and trafficking of NPRA remains to be determined (Table 2). Dileucine (LL) motifs regulate internalization and trafficking of various membrane receptors through the endocytic and secretory pathways [27,180,192,223,224,225]. LL motifs are characterized by four to seven amino acid residues in which LL residues are usually preceded by a polar residue and a negatively charged amino acid residue, which may be aspartic acid, glutamic acid, or phosphoserine. Although, dileucine motifs with acidic amino acids are constitutively active, serine residues in LL motifs are activated by phosphorylation [223]. LL motifs regulate both endocytosis and secretory pathways of membrane receptors and proteins [226,227]. In the cytoplasmic tail of GABA receptor, LL motifs can act at the level of TGN to control the receptor expression on the cell surface and also serve as endocytic signals [227,228,229]. Both LL- and tyrosine-based signals interact with clathrin-associated AP complexes, which help to recruit membrane receptor into clathrin-coated vesicles. Dileucine motifs can bind to mu subunits of APs, but some [DE] xxxL[LI] motifs interact with gamma and sigma 1 subunits of AP-1 and AP-2, as well as with delta and sigma three subunits of AP-3 [230,231,232]. The dileucine-based signal motifs also play roles in vesicular transport of membrane proteins, including acetylcholine transporter (VAchT), GLUT 1 (VGLUT1), and tyrosinase [192,233,234]. Some membrane receptors contain more than one LL motif; one of the leucine residues can be substituted for tyrosine-based signal motifs. In CD3 and mannose-6-phosphate receptors, LL motifs correspond to a distinct class of signal that includes [DE] xxxL[LI] and DxxLL motifs. These features of dileucine-type signal motifs suggest that they can be recognized at the plasma membrane and also in the intracellular compartments [192].

It has been previously suggested that DxxLL signal motifs also participate in recycling of membrane proteins between the TGN and endosomes [27]. [D/E]xxxL [L/I] signal motifs interact and bind to mu as well as beta subunits of APs [235,236,237,238]. Crystallographic studies have indicated that AP-2 adaptor core binds to LL motif and interacts with AP-2 complex [239]. LL-based sorting motifs in mannose-6-phosphate receptors are recognized by ADP-ribosylation-factor (ARF)-dependent clathrin adaptor proteins, which are referred to as Golgi-localizing gamma-adaptor homology domain ARF-binding proteins (GGAs) and display critical roles in the packaging of mannose-6-phosphate receptor into clathrin-coated vesicles in the TGN compartments [182,240,241]. To ascertain the function of LL-based signals in relation to [D/E]xxxL [L/I] or DxxxL motifs, it should be noted that the former is recognized by heterotrimeric adaptor complexes such as AP-1, AP-2, AP-3, and AP-4, which bind GGA adaptor proteins [241,242,243,244]. Although, dileucine motifs bind to AP-1, AP-2, AP-3, and AP-4 complexes, the binding characteristic of a complex seem to differ from the specific recognition of a diverse repertoire of LL signals [225,245]. However, much investigation is needed regarding the role of LL signal motifs in the internalization of NPRA.

A number of short sequence signal motifs are located in the ligand-binding domain, protein-kinase-like homology domain (Protein-KHD), and guanylyl cyclase (GC) catalytic domain of NPRA [5]. However, signal motifs located in the extracellular domains of membrane receptor, are not expected to direct internalization. NPxY motif is well known to promote the internalization of various membrane receptors; however, due to its location in the ligand-binding domain, it is not anticipated to participate in the internalization of NPRA. Thus far, only GDAY motif has been reported to participate in the internalization and trafficking of NPRA [26]. However, the role of other putative motifs present in the protein-KHD and GC catalytic domain of NPRA, remains to be determined [5].

### 4.4. YXXphi-Type Signal-Sequence Motifs and Internalization of Membrane Receptors

Interestingly, Yxxphi motif is located in the carboxyl-terminal domain of NPRA at residues Y^988^-x-x-F^991^; however, its role in the internalization of NPRA has yet to be determined. Usually, Yxxphi signal motifs with internalization specificity are located within 10–40 amino acid residues from the transmembrane domain of various membrane receptors [245,246,247]. The tyrosine-based Yxxphi sorting signals direct the internalization of receptors by interacting with mu1, mu2, mu3, and mu4 subunits of adaptor proteins, including AP-1, AP-2, AP-3, and AP-4, respectively [201,248,249]. The tetrapeptide sequence Yxxphi is usually located in the cytoplasmic domains of several transmembrane receptors such as transferrin and asialoglycoprotein receptors and plays critical roles in the internalization process. In Yxxphi signal motifs, Y is a tyrosine residue, x is any amino acid residue, and phi is an amino acid residue with large bulky hydrophobic side chain. Yxxphi signal motifs exhibit dual specificity, such as an endocytotic functional motif and a trafficking signal within the endosomal and/or secretory pathways [27,37,178,245]. As mentioned above, the adaptor protein complexes sort the cargo in the coated vesicles for trafficking of receptors from one subcellular compartment to another location inside the cell.

A new member of AP complex, AP-5 seems to participate in the endosomal sorting of membrane receptors [250]. In particular, Yxxphi-based signals mediate sorting of transmembrane receptors into endosomes and basolateral plasma membranes [34]. The Y residue is critical for endocytic signals and it cannot be substituted by other aromatic residues, since the phenolic hydroxyl group of the tyrosine is essential for generating the endocytic and trafficking signals. The two x residues also seem to contribute to the specificity and potency of signals for both endocytic and sorting events. The phi position in tetrapeptide sequence accommodates a varying number of amino acids containing bulky hydrophobic side chains [251,252]. The identity of amino acid residues at phi position confers the specificities of trafficking and sorting signals. However, both tyrosine and phi residues of Yxxphi motifs are important in internalization and trafficking events of membrane receptors.

The tetrameric Yxxphi motifs also play roles in the lysosomal targeting, which are usually located at six to eight amino acid residues from the transmembrane domain in the cytoplasmic region of membrane receptors, contain acidic residues at the x position [27,251]. In certain circumstances, in Yxxphi motif, a glycine residue precedes the tyrosine residue (YGxphi). The substitution of alanine in the place of glycine interferes with lysosomal targeting, but does not affect the endocytotic processes [253]. However, the presence of a glycine residue before the critical tyrosine residue of Yxxphi motifs helps to recognize lysosomal membrane proteins [242]. The YSGL motif interacts with currently unknown intracellular proteins and governs the constitutive internalization of chemokine-CxCR3 receptors [254]. Previous studies have suggested that Yxxphi motifs transmit signals similar to YKKL motifs within the carboxyl-terminal domain of protease-activated receptor-1 (PAR-1), which regulate the clathrin- and dynamin -dependent internalization processes [201]. YxxGL motifs also direct the internalization and sorting of P_2_X_4_ receptors [255]. Similarly, the VxxSL motif seems to be critical for cell surface expression of voltage-gated K^+^ channels [191]. It has also been shown that a HLVNK motif plays a dual role either as a TGN sorting signal for the localization of CD44 in the basolateral membrane or as an internalization motif necessary for transcytosis of CD44 in the apical membrane [256,257,258]. Interestingly, GTALL motif has been shown to direct the trafficking of luteinizing hormone receptor from the degradative pathway to recycling process [187]. The previous studies have suggested that residues in the conserved regions of receptors might play a pivotal role in the internalization and trafficking processes [38]. The molecular mechanisms by which the large sequence regions of various receptors signal during the internalization and trafficking processes, still remain elusive and not yet clearly understood.

## 5. Conclusions

NPRA is a multifunctional receptor molecule, which constitutes ANP-binding activity, transmembrane spanning ability, protein-KHD autoregulatory properties, and GC catalytic activity. The intracellular regulation of NPRA is governed by its movement through the multiple subcellular compartments inside the cell. It is implicated that two major components direct the proper trafficking and sorting of NPRA. First, the interaction of NPRA with its ligands (ANP and BNP) modulates the kinetic rates at which receptors move through the subcellular compartments. Second, NPRA itself is a major determinant, which systematically directs proper routing in the intracellular compartments. The kinetic rate at which receptor traverses in the intracellular compartments influences the sensitivity of cells to its ligands. Substantial evidence supports the notion that the expression and cellular regulation of membrane receptors is accomplished by their insertion on the plasma membrane, ligand binding characteristics, and movement of ligand-receptor complexes through coated vesicles into the cell interior. Kinetics of stoichiometric distribution of ligand-bound receptors from plasma membrane to intracellular compartments has provided definitive means of determining the dynamics of ligand-mediated translocation and redistribution of NPRA in intact cells. In this process, ligand-bound NPRA is rapidly internalized and delivered to endosomes, while a majority of ligand-receptor complexes are degraded in lysosomal compartments; however, a small population of receptors seems to be dissociated from ligands in the intracellular compartments and recycles back to the plasma membranes.

Small-sequence signal motifs play critical roles, which inherently control internalization, trafficking, and redistribution of ligand-receptor complexes in the intracellular compartments at the molecular level. The internalization and trafficking signals for cargo recognition into coated vesicles, are usually present in the form of short linear sequences, which play critical roles in the specific routing pathways that might be signature-specific to cell types for different receptors, including NPRA. The cellular regulation and expression of NPRA involve internalization, trafficking, and movement of ligand-activated receptors into the subcellular compartments. The receptor biosynthesis and subcellular assembly that might be responsible for receptor expression and function also remain to be defined in a cell-specific manner. Future research needs to be directed towards the biosynthetic assemblies and regulatory functions of NPRA endocytosis, trafficking, desensitization, down-regulation, and metabolic degradation along with other members of natriuretic peptide receptors and also GC family of receptors. In addition, investigations are needed to underscore the molecular basis of the endocytic processes, as well as its relationship with NPRA phosphorylation, dephosphorylation, and/or glycosylation in the context of internalization, trafficking, down-regulation and/or desensitization in subcellular compartments.

## Definitions

ANP: atrial natriuretic peptide
BNP: brain natriuretic peptide
CNP: C-Type natriuretic peptide
GC-A/NPRA: guanylyl cyclase/natriuretic peptide receptor-A
GC-B/NPRB: guanylyl cyclase/natriuretic peptide receptor-B
NPRC: natriuretic peptide clearance receptor
KHD: kinase homology domain
GC: guanylyl cyclase
EGF: epidermal growth factor
HEK-293cells: human embryonic kidney-293 cells
*Npr1*: guanylyl cyclase/natriuretic peptide receptor-A gene
miRNA: microRNA
pCMV: plasmid with cytomegallovirus promoter
TGF-βR: transforming growth factor-beta receptor
Dab-1/2: disabled-1/2
AP-1-5: adaptor protein-1-5
PTB: phosphotyrosine binding
LDL: low density lipoprotein
GABA: γ -aminobutyric acid
ARF: ADP-ribosylation factor
TGN: trans-Golgi network
GGA: Golgi-localizing gamma-adaptor
